# Electricity in Dental Surgery

**Published:** 1876-06

**Authors:** Geo. H. Perine

**Affiliations:** New York.


### ARTICLE II.

### *Electricity in Dental Surgery.*

BY GEO. H. PERINE, D. D. S., NEW YORK.

The value of Electricity as a therapeutic agent has been long conceded, and its application in morbid pathology has been frequent and satisfactory. Its influence in diseases of the nervous system, is marked and peculiar, and is certainly capable of a degree of development in judicious hands, even far beyond the progress which it has already made. Naturally this powerful agent could not have failed at one time or another to be brought into use in the practice of dental surgery. That this has not been the case to a greater extent than is the fact, is owing perhaps chiefly to neglect on the part of the members of the profession to avail themselves of an agent whose value has been long appreciated by their medical brethren. Of late, however, the attention of dentists has been directed towards the employment of Electricity in their practice, and with the best results.

Among the diseases which occur in dental practice, and which may properly be treated by that form of Electricity developed by the galvanic battery, may be mentioned, *Neuralgia*, *Tetanus*, *Grandular Swellings*, *Tic Douloureux*, *Palsy of the Face*, *Facial Paralysis*, *&c.* While used as a motive power to vitalize the instrument in Galvano Cautery, it may be employed to advantage to sever diseased growths from contiguous healthy tissue, and to cauterize unhealthy ulcers, sinews, &c. Frequently in dental operations, it becomes necessary to amputate diseased parts of the mouth or malignant growths, which can not be conveniently reached with the knife, and which from location or otherwise are difficult to treat, and control the hemorrhage from vessels that cannot be conveniently ligatured. This mode of practice, aided by the proper instruments may be used with the most satisfactory and important results. Allen McLane Hamilton, M. D., New York, employs galvanism with great success in cases of Facial Neuralgia. Dr. Alfred L. Carroll, New Brighton, employs galvanism for Facial Neuralgia. He places the poles over the nucha or lower down, and over the mastoid bone, or upon both temples. For Neuralgia we have had satisfactory results, by applying the positive pole at the back of the ear, and the negative passed over the several branches of the fifth nerve, commencing with a mild current, and increasing if the case be violent. Dr. Caldwell, Baltimore, gives interesting cases showing the effects of Electricity. Dr. John Butler in his experience in *Galvanic Surgery*, refers to a case in which he operated for a nævus of the lower lip. In this case the growth was entirely removed by a single operation, made with three needles connected with a small battery; in three days a small dry scab had taken the place of the nævus, and in two weeks there was no mark whatever, nor anything to indicate that there ever had been any abnormal growth. In the case of sensitive dentine, the Galvano Cautery may be applied with perfect safety, and with the most happy effect. The application of the instrument in such a case when properly employed, will destroy the sensibility to pain, and thus materially facilitate the after operation

A case of Facial Neuralgia is reported by Dr. Jos. Stead, Manchester. The patient was a girl 24 years of age, who had been suffering from Facial Neuralgia of both sides for three months; ten minutes application of Electricity entirely cured the patient, and there was not the slightest return of n. A very important use of this agent may be made by the dentist in its application for cauterizing nerves of the teeth when exposed, also treating of alveolar abscess and hemorrhage after extraction of teeth, &c.

Joseph Snape, L. D. S., R. C. S., Liverpool, recommends, Electricity as a means of producing anæsthesia for dental operations. He offers every facility to the profession to prove for themselves the usefulness of this agent, which he has found to be effectual. The employment of this important remedial agent, has been of late so greatly facilitated by the invention of new and improved batteries, which are compressed in a small compass and are readily portable, that no practitioner need be deterred from its use by fear of complicated mechanism. A simple little instrument is furnished by the manufacturer, which may be carried in the hand to the chair, or the bed side of the patient, and the application made without pain, trouble, or loss of time. By the judicious use of such means as we have indicated, the practice of dental surgery becomes emendated, and is shorn of half its terrors and all its danger.

